# Role of the mitochondrial regulatory factor CHCHD2 in neurodegenerative diseases

**DOI:** 10.3389/fnins.2025.1639651

**Published:** 2025-09-02

**Authors:** Xinyu Guo, Peiyu Xu, Chen Liang, Yuntao Li

**Affiliations:** ^1^Department of Neurology, Second Affiliated Hospital of Nanjing Medical University, Nanjing, China; ^2^Department of General Practice, Second Affiliated Hospital of Nanjing Medical University, Nanjing, China

**Keywords:** CHCHD2, mitochondria, structural and functional abnormalities, mutation, neurodegenerative disorders

## Abstract

Mitochondria are essential organelles within cells, and their dysfunction is associated with many neurodegenerative disorders. The protein CHCHD2, which is situated in the intermembrane space of mitochondria, plays a pivotal role in mitochondrial function. Its knockdown or mutation is linked to mitochondrial impairment. Although research suggests that CHCHD2 is involved in the mechanisms underlying various neurodegenerative diseases, there is a notable absence of comprehensive studies that integrate different mutation types, pathogenic mechanisms, and targeted treatment strategies. This paper provides a review of CHCHD2’s structure and function, mutant varieties, biological models, and relevant therapies. We conclude that CHCHD2 is critical for maintaining mitochondrial homeostasis, facilitating cell migration, and regulating apoptosis. Mutations in CHCHD2 may influence the mechanisms of neurodegenerative diseases through both loss-of-function and gain-of-function effects, with overexpression possibly reversing pathological processes and mitochondrial dysfunction. Furthermore, elamipretide, a novel drug that targets mitochondria, has shown efficacy in partially alleviating mitochondrial defects resulting from CHCHD2 mutations. These insights could inform the identification of therapeutic targets in neurodegenerative diseases and shape future research on CHCHD2.

## 1 Introduction

Neurodegenerative diseases (NDDs) are a heterogeneous group of neurological disorders characterized by the progressive loss of neurons and the deterioration of neural network structure and function. This decline ultimately leads to impairments in memory, cognition, behavior, sensation, and/or motor function (Wilson et al., 2023). Notable examples of NDDs include Alzheimer’s disease (AD), Parkinson’s disease (PD), Huntington’s disease (HD), frontotemporal dementia (FTD), Lewy body dementia (LBD), and amyotrophic lateral sclerosis (ALS), among others. The prognosis for individuals diagnosed with these conditions is generally poor, significantly impacting their quality of life. Consequently, developing effective treatments for NDDs presents a major challenge in the field of medicine.

Mitochondria are essential organelles found in eukaryotic cells, playing a pivotal role in various cellular activities such as energy metabolism, programmed cell death (apoptosis), signal transduction, and the metabolic pathways related to lipids, amino acids, and iron (Chacinska et al., 2009). The structure of mitochondria comprises the inner mitochondrial membrane (IMM), the outer mitochondrial membrane (OMM), and the matrix. Within the matrix reside enzymes responsible for intermediate metabolism and the mitochondrial genome, which encodes certain proteins and RNA necessary for their translation (Frey and Mannella, 2000). While most mitochondrial proteins are encoded by the nuclear genome and synthesized as precursor proteins in cytoplasmic ribosomes, they are imported into mitochondria through multiple mechanisms to contribute to diverse physiological functions (Chacinska et al., 2009). Mutations or dysfunctions in mitochondrial proteins can significantly contribute to mitochondrial dysfunction. Given that the mitochondrial energy supply is crucial for various cellular activities, dysfunction can result in cellular damage, particularly affecting energy-demanding cells like neurons and muscle cells. Current research indicates that mitochondrial dysfunction plays a role in the pathogenesis of several neurodegenerative diseases, including Alzheimer’s disease, frontotemporal dementia, Parkinson’s disease, motor neuron disease, and mitochondrial myopathy, and is also closely linked to apoptosis and cancer.

Coiled-Coil-Helix-Coiled-Coil-Helix Domain Containing 2 (CHCHD2) is a member of the eukaryotic twin cysteine-X9-cysteine (CX9C) protein family and functions as a mitochondrial metabolic regulator. It is primarily localized in the intermembrane space of mitochondria, with some presence in the cell nucleus (Koehler and Tienson, 2009; Aras et al., 2015; Gladyck et al., 2021). Research indicates that mutations in the CHCHD2 gene may contribute to the pathogenic mechanisms underlying neurodegenerative diseases such as Parkinson’s disease, frontotemporal dementia, and Alzheimer’s disease, and are also associated with apoptosis and cancer. This review summarizes and discusses the structure and function of CHCHD2, its links to neurodegenerative diseases, and current targeted mitochondrial therapeutic strategies.

## 2 Structure and function of CHCHD2

### 2.1 Structure and localization of CHCHD2

CHCHD2 is a member of the eukaryotic twin CX9C protein family, characterized by the CHCH structural domain. This domain features a convoluted CHCH fold, which is connected by four cysteines that form two stable disulfide bonds, referred to as the CX9C motif, known for its evolutionary conservation (Gladyck et al., 2021). The human CHCHD2 protein consists of 151 amino acids, is soluble, and its encoding gene is located on chromosome 7 (7P11.2; Modjtahedi et al., 2016). The protein contains a positively charged N-terminal motif with several arginine residues, a hydrophobic central *α*-helix, and a C-terminal CHCH structural domain (Shammas et al., 2023). Under normal physiological conditions, CHCHD2 is encoded by nuclear genes, synthesized in the cytoplasm, and then transported to the mitochondrial intermembrane space (IMS) via its CX9C motif, where it binds to Mia40 anchored to the inner membrane and is subsequently transported through the Mia40/Erv1 disulfide bond relay system (Aras et al., 2015; Gladyck et al., 2021; Modjtahedi et al., 2016; Shammas et al., 2023; Chacinska et al., 2004). In contrast, under stress conditions, the transport of CHCHD2 into the mitochondria is inhibited, resulting in increased accumulation of the protein in the nucleus, which enhances its transcriptional function (Aras et al., 2020).

### 2.2 Functions of CHCHD2

CHCHD2, as a mitochondrial metabolic regulator, performs a variety of functions in the mitochondria of various cells.

#### 2.2.1 Participates in oxidative phosphorylation and stabilizes mitochondrial cristae structure

Baughman et al. (2009) predicted novel regulators of oxidative phosphorylation through expression screening and found that silencing the CHCHD2 gene resulted in a significant reduction in cellular oxygen consumption rates and a marked defect in oxidative phosphorylation. This led to the conclusion that CHCHD2 may play a role in the regulation of oxidative phosphorylation by influencing cytochrome c oxidase (COX, complex IV). This finding was corroborated by other studies (Aras et al., 2017). Additionally, Aras et al. (2013) demonstrated that CHCHD2 acts on the oxygen-responsive element located in the proximal promoter of COX4I2 as a transcription factor, regulating the hypoxic expression of COX4I2, which in turn affects COX activity. Under 4% oxygen, CHCHD2 not only functions as a key transcription factor for COX4I2 but is also transcriptionally stimulated by hypoxia, thereby activating its own positive feedback loop and promoting the production of its transcripts and proteins (Aras et al., 2015). This phenomenon has been reported in other studies as well (Aras et al., 2020). A recent study indicated that CHCHD2 induces structural changes in helix X within the heme periphery to accelerate proton uptake (Yanagisawa et al., 2024). Furthermore, knockdown of CHCHD2 reduced cellular ROS scavenging (Aras et al., 2015), likely due to dysfunction of cytochrome c oxidase induced by CHCHD2 deficiency, which leads to ROS accumulation and increased oxidative stress, underscoring its crucial role in maintaining the cellular redox state.

Most of the mitochondrial oxidative phosphorylation processes occur at the mitochondrial cristae, which are folds of the inner mitochondrial membrane. CHCHD2 not only regulates oxidative phosphorylation but also plays a crucial role in stabilizing mitochondrial cristae. Deficiency of CHCHD2 disrupts the membrane organization system of the mitochondrial inner membrane (MICOS), adversely affecting mitochondrial morphology, function, and cristae structure (Lu et al., 2022). The role of CHCHD2 in maintaining mitochondrial cristae stability has been corroborated by other studies (Zhou et al., 2019) and confirmed through *in vivo* experiments in mice (Liu et al., 2020) and Drosophila (Meng et al., 2017), indicating its significant contribution to the integrity of mitochondrial cristae morphology. Recent research has further supported this notion, highlighting that mitochondrial dysfunction resulting from MICOS disruption is a key factor in ciliary dysfunction in astrocytes (Leventoux et al., 2024). However, the molecular mechanisms by which CHCHD2 preserves mitochondrial cristae stability remain unclear and warrant further investigation.

#### 2.2.2 Promotes cell migration and regulate apoptosis

The expression of CHCHD2 influences not only the morphology and function of mitochondria but also correlates with cell migration and apoptosis. Seo et al. (2010) demonstrated that the overexpression of CHCHD2 promotes cell migration, while its knockdown has the opposite effect, as revealed through functional genetic screening of novel cell migration-promoting genes. This suggests that CHCHD2 may serve as a determinant gene for cell migration. Moreover, CHCHD2 has been implicated in the regulation of cell migration and angiogenesis in both renal cell carcinoma (Cheng et al., 2019) and hepatocellular carcinoma (Yao et al., 2019).

In response to cellular stress, mitochondria can induce endogenous apoptosis through mitochondrial outer membrane permeabilization (MOMP), a process primarily regulated by the B-cell lymphoma 2 (Bcl-2) protein family (Chipuk et al., 2010). Research indicates that CHCHD2 regulates apoptosis by interacting with various members of the Bcl-2 family (Liu Y, et al., 2015). The progression of cancer is largely attributed to the unrestricted proliferation of cancer cells, enhanced migratory capabilities, and reduced apoptosis. Thus, the interaction of CHCHD2 with Bcl-2 family members may be instrumental in promoting cell proliferation and invasion while inhibiting apoptosis (Yao et al., 2019). Furthermore, the role of CHCHD2 in apoptosis prevention has been confirmed in adrenal tumors (Karapanagioti et al., 2023), non-small cell lung cancer (Yin et al., 2020; Wei et al., 2015), glioblastoma (Lumibao et al., 2023), and breast cancer (Ma et al., 2020), although the mechanisms of action vary among these contexts. It is concluded that the regulation of cell migration, proliferation, and apoptosis by CHCHD2 is crucial to its oncogenic effects; however, the specific molecular mechanisms underlying CHCHD2-mediated oncogenesis remain unclear. Recently, some scholars suggested that mitochondrial autoimmunity is a major factor in breast cancer development by reviewing the studies on mitochondrial autoimmunity and breast cancer development, and CHCHD2 can mediate mitochondrial function to promote the proliferation and migration of cancerous cells, which in turn supports the validation of the involvement of mitochondrial autoimmunity in carcinogenesis (Madrid et al., 2020). Nonetheless, evidence supporting this concept from other studies is limited, necessitating further exploration and validation.

In the nervous system, CHCHD2 is expressed at high levels in neurons, particularly in midbrain dopaminergic neurons (Nguyen et al., 2022). This suggests that dopaminergic neurons have a greater dependency on CHCHD2 and may therefore be more susceptible to mutations in this gene. CHCHD2 mutations are closely associated with Parkinson’s disease (PD), with the primary mechanism involving the preferential degeneration of midbrain dopaminergic neurons. As a critical regulator of mitochondrial function, CHCHD2 plays an essential role in neurons by modulating mitochondrial energy metabolism and responses to oxidative stress. Since neurons are highly sensitive to mitochondrial health, abnormalities or deficiencies in CHCHD2 can easily lead to neuronal damage and cell death, thereby contributing to the onset and progression of neurodegenerative diseases.

## 3 CHCHD2 and neurodegenerative diseases

### 3.1 Mutant subtypes of the CHCHD2 gene

In 2015, CHCHD2 was identified for the first time to be significantly associated with inherited Parkinson’s disease. Subsequently, extensive research further elucidated the role of this gene, revealing that it not only plays an important part in the pathogenesis of Parkinson’s disease but also shows correlations with other neurodegenerative disorders. Given that the underlying mechanisms of neurodegenerative diseases often involve mitochondrial dysfunction, researchers have conducted genetic analyses across diverse populations and family lineages worldwide. These studies aim to systematically investigate the pathogenic role of CHCHD2 in various neurodegenerative conditions (Table 1).

**Table 1:** The mutation of CHCHD2 in various populations.

Country	Mutation	Disease	References
Japan	182C>T (T61I); 434G>A (R145Q); 300 + 5G>A	PD	Funayama et al. (2015)
Japan	c.23G>A (p. R8H)	PD	Ikeda et al. (2017)
Japan	8 T>G; c.41C>T (p. P14L)	ALS	Ikeda et al. (2024)
China	T61I; P2L	PD	Shi et al. (2016)
China	P2L	PD	Li et al. (2016)
China	P2L	PD	Liu and Li (2015)
China	A79S	PD	Yang et al. (2019)
China	P2L	ET	Wu et al. (2016a)
China	P2L; 238A>G (I80V)	AD	Liu et al. (2018)
China	P2L; c.15C>G (p. S5R); c.94G>A (p. A32T)	AD	Che et al. (2018)
China	P2L; c.255 T>A(p. S85R)	FTD	Che et al. (2018)
China	P53fs	FTD	Nan et al. (2024)
America; Poland; Ireland	p. P2L; p. G4R; p. P14S; p. A16A; p. V31V; p. P34L、p. A37V; p. A49V; p. A93V	PD; LBD	Ogaki et al. (2015)
Germany	c.376C>T (p. Gln126X)	PD	Koschmidder et al. (2016)
Western Europe	Ala32Thr; Pro34Leu; Ile80Val	PD	Liu and Li (2015)
Italy	c.196G>A (p. Val66Met)	MSA	Nicoletti et al. (2018)
Australia	c.211G>C (p. Ala71Pro)	PD	Lee et al. (2018)

#### 3.1.1 Japanese

Funayama et al. (2015) conducted a genome-wide linkage analysis in 2015 on a Japanese family with autosomal dominant Parkinson’s disease (ADPD), establishing for the first time the association between the CHCHD2 gene and Parkinson’s disease. This study was the first to report three CHCHD2 mutations: c.182C > T (T61I), c.434G > A (R145Q), and c.300 + 5G > A. Additionally, two single nucleotide variants, 9 T > G and 5C > T, were identified. Subsequently, Ikeda et al. (2017) detected a novel mutation, c.23G > A (p. P8H), within the mitochondrial targeting sequence in Japanese PD patients, further confirming the association of CHCHD2 with Parkinson’s disease in this population. Furthermore, Ikeda et al. (2024) identified two new mutations, 8 T > G and c.41C > T (p. P14L), in genetic analyses of patients with amyotrophic lateral sclerosis (ALS).

#### 3.1.2 Chinese

Building upon the research conducted by Liu Z, et al. (2015) performed a comprehensive screening of familial autosomal dominant Parkinson’s disease (ADPD) cases in mainland China in the same year. However, they did not identify any CHCHD2 mutations. Subsequently, further studies on the Chinese PD population have revealed various gene mutations, including the T61I mutation (Shi et al., 2016), P2L mutation (Shi et al., 2016; Li et al., 2016; Liu and Li, 2015), and a rare variant p. A79S (Yang et al., 2019). In addition, the c.5C > T (P2L) mutation was found in patients with idiopathic tremor (Wu et al., 2016a). Among Alzheimer’s disease (AD) patients, reported mutations include P2L (Liu et al., 2018; Che et al., 2018), c.238A > G (I80V; Liu et al., 2018), c.15C > G (p. S5R; Che et al., 2018), and c.94G > A (p. A32T; Che et al., 2018). In patients with frontotemporal dementia, mutations such as P2L, c.255 T > A (p. S85R; Che et al., 2018), and a recently identified p. P53fs mutation (Nan et al., 2024) have been observed. Although multiple studies suggest an association between CHCHD2 and neurodegenerative diseases in the Chinese population, some research indicates that CHCHD2 may not play a key role in the pathogenesis of neurodegenerative diseases (Yang et al., 2016; Fan et al., 2016; Wu et al., 2016b; Lu et al., 2016). The discrepancies among results across different studies could be attributed to factors such as geographic region, family lineage, and the heterogeneity of disease mechanisms.

#### 3.1.3 Other populations

Nine rare exon variants were also discovered in studies involving PD families and frontotem-poral lobe dementia families in the United States, Poland, and Ireland, including p. P2L, p. G4R, p. P14S, p. A16A, p. V31V, p. P34L, p. A37V, p. A49V, and p. A93V. These findings suggest that CHCHD2 mutations may be associated with the risk of developing PD and Lewy body dementia (LBD) in these populations (Ogaki et al., 2015). In a German PD population, Koschmidder et al. (2016) similarly identified a pathogenic mutation (c.376C. T, p. Gln126X) located within the structural domain of CHCH. In the PD population of Western Europe, three mutants were found, namely Ala32Thr, Pro34Leu and Ile80Val (Liu and Li, 2015). Another novel mutation, c.196G > A (p. Val66Met), was found in an Italian study of patients with multiple system atrophy, indicating a potential association between CHCHD2 and the pathogenesis of this disease (Nicoletti et al., 2018).

In contrast, patients with mitochondrial myopathy in Italy showed no association with CHCHD2 (Rubino et al., 2018). An exon missense mutation, c.211G > C (p. Ala71Pro), was detected in a female patient with early-onset PD in Australia, suggesting that CHCHD2 may be involved in the pathogenesis of recessive early-onset PD (Lee et al., 2018). However, no association between CHCHD2 and the disease has been identified in the Brazilian (Voigt et al., 2019), Spanish (Parrado et al., 2017) and Sweden (Liu and Li, 2015) PD populations.

In summary, the role of CHCHD2 mutations in the pathogenesis of neurodegenerative diseases remains a topic of debate, influenced by racial, geographic, and familial factors. While the types of CHCHD2 mutations vary across different diseases and populations, a significant association between this gene and neurodegenerative disease pathogenesis appears likely. However, due to variations in ethnicity, geography, lifestyle, and genetic profiles among affected populations, as well as the complex nature of neurodegenerative diseases characterized by a wide range of mutations, we hypothesize that CHCHD2 may contribute to disease etiology in only a subset of patients. This contribution may result from the synergistic effects of multiple genes rather than CHCHD2 being the sole causative factor. Further studies are essential to examine the genetic landscape of affected populations, which will help validate the relationship between CHCHD2 and neurodegenerative diseases and support its consideration as a potential therapeutic target.

### 3.2 Cellular and animal models associated with CHCHD2

Since the discovery in 2015 that CHCHD2 mutations are associated with neurodegenerative diseases, researchers have sought to validate the pathogenic mechanisms mediated by these mutations through the establishment of cellular and animal models. In 2017, Meng et al. (2017) first intro-duced the T61I and R145Q mutants, along with normal CHCHD2, into a Drosophila model with deficient CHCHD2 expression to investigate its pathogenesis. Their findings indicated that these mutants could not rescue mitochondrial dysfunction caused by CHCHD2 deletion, supporting the conclusion that CHCHD2 mutations may lead to disease through a loss-of-function mechanism. Subsequently, Zhou et al. (2019) discovered that mutations in models of human embryonic stem cells and neural progenitor cells (carrying R145Q and Q126X mutations) led to a reduction in mitochondrial cristae and impaired MICOS complex function, phenotypically similar to CHCHD2 knockout models. This finding further supports the hypothesis. The studies suggest that the loss of function of mutant proteins may mediate disease progression through several mechanisms: (1) abnormal aggregation forming insoluble polymers (Meng et al., 2017; Cornelissen et al., 2020; Liao et al., 2024; Kee et al., 2022; Huang et al., 2019); (2) failure to correctly localize to the mitochondrial cristae membrane (Mao et al., 2019), resulting in collapse of cristae structure; (3) impaired respiratory chain function; and (4) induction of apoptosis (Zhou et al., 2019; Meng et al., 2017; Mao et al., 2019).

However, many studies have also pointed out that the mechanisms underlying CHCHD2 mutations are complex and may involve not only loss-of-function but also gain-of-function mechanisms. Harjuhaahto et al. (2020) found that although CHCHD2 knockout impairs cellular respiration and synaptic function, human induced pluripotent stem cells (iPSCs) can survive and successfully differentiate into functional motor neurons *in vitro*. This suggests that mutant CHCHD2 proteins may promote disease through gain-of-function mechanisms. Similarly, in a mutant mouse model (T61I) established using the knock-in technique by Fan et al. (2023), key clinical and neuropathological features characteristic of Parkinson’s disease (PD) were reproduced, and the insulin-degrading enzyme (IDE) was identified as potentially involved in the pathogenesis of mitochondrial dysfunction induced by the CHCHD2 mutation in aged mice. This study suggests that the mutant and the deletion of CHCHD2 are not functionally equivalent, and there may be another mechanism. Subsequently, Kee et al. (2022) developed the first T61I mutation transgenic mouse model driven by the mPrP promoter, which effectively mimicked the neurodegenerative features of PD. Their findings showed that the T61I mutation significantly affected oxidative phosphorylation, mitochondrial function, and multiple metabolic pathways and protein expressions associated with neurodegeneration, while also promoting *α*-synuclein aggregation. Notably, the progression of phenotypic features in this mutant model was considerably faster than in knockout models, contrasting with the results reported by Sato et al. (2021). Furthermore, Nguyen et al. (2022) observed no similar pathological changes in CHCHD2 knockout mice, further suggesting that CHCHD2 mutations may involve a toxic gain-of-function rather than a simple loss. Additionally, Torii et al. (2023) found that in Neuro2a cells and dopaminergic neurons of mice harboring the T61I mutation, the mutant protein was abnormally localized in the cytoplasm. It recruited casein kinase 1 epsilon/delta (Csnk1e/d) to phosphorylate neurofilaments and *α*-synuclein, leading to the formation of cytoplasmic aggregates. Inhibition of Csnk1e/d alleviated cell injury, indicating that these kinases may play a crucial role in mutation-induced PD, providing new evidence for a gain-of-function pathogenic mechanism. Moreover, Liao et al. (2024) reported elevated *α*-synuclein levels in T61I mutant mice, and their genomics and proteomics analyses further confirmed that the mutation exerts a toxic gain-of-function effect. In related research, Chen et al. (2024) demonstrated that in an MPP^+^-induced PD model, overexpression of the T61I mutant led to decreased F1F0-ATPase activity, promoted mitochondrial permeability transition pore (mPTP) opening, increased mitochondrial permeability, and exacerbated neuronal degeneration and behavioral deficits. Collectively, these studies suggest that toxic gain-of-function mechanisms may play a key role in CHCHD2 mutation-induced neurodegenerative diseases.

Some studies have shown that the mutant CHCHD2 may cause pathogenic effects in cells through two different mechanisms. In the study of human fibroblasts with T61 I mutation, Cornelissen et al. (2020) found that the mutant has a dual role: on the one hand, the mutant protein can not play its normal function due to the deposition in the mitochondria, resulting in abnormal oxidative respiration of the mitochondria and increasing the production of ROS. This mechanism is consistent with the model of functional loss; on the other hand, the mutant protein can induce apoptosis after entering the mitochondria (while the mutant in the cytoplasm alone has no such effect), and can induce wild-type CHCHD2 misfolding and deposition, reflecting the mechanism of functional acquisition. Similarly, Mao et al. (2019) discovered that in SH-SY5Y neuroblastoma cells, the T61I mutation not only results in functional loss but also promotes aberrant binding to CHCHD10. This interaction causes CHCHD10, normally localized to mitochondria, to be misretained in the cytoplasm, thereby exacerbating mitochondrial dysfunction. These findings confirm that CHCHD2 mutations may contribute to neurodegenerative disease through both loss-of-function and gain-of-function (toxic) mechanisms.

Drawing on these various studies of CHCHD2, we hypothesize that there are both loss-of-function effects relative to the normal protein and a toxic gain of function due to mutation (Table 2). However, the precise molecular mechanisms remain unclear. Given the complexity of the pathogenic mechanisms underlying neurodegenerative diseases, which may involve synergistic interactions among multiple genes, there is a need to establish more representative mutation models to investigate these potential molecular mechanisms further.

**Table 2:** The mutation model and supporting mechanism of CHCHD2.

Number	Mutation	Models	Mechanisms	Disease	References
1	T61I; R145Q	Fruit flies	loss-of-function	PD	Meng et al. (2017)
2	R145Q; Q126X	human embryonic stem cells and neural progenitor cells	loss-of-function	PD	Zhou et al. (2019)
3	T61I	mice	gain-of-function	PD	Fan et al. (2023)
4	T61I	mice	gain-of-function	PD	Kee et al. (2022)
5	T61I	neuro2a cells and mice	gain-of-function	PD	Torii et al. (2023)
6	T61I	mice	gain-of-function	PD	Liao et al. (2024)
7	T61I	SH-SY5Y cell	gain-of-function	PD	Chen et al. (2024)
8	T61I	human fibroblasts	loss-of-function and gain-of-function	PD	Cornelissen et al. (2020)
9	T61I	SH-SY5Y cell	loss-of-function and gain-of-function	PD	Mao et al. (2019)

Few studies have examined the role of CHCHD2 mutations in the pathogenesis of other neurodegenerative diseases. Recently, Ikeda et al. (2024) investigated the pathogenic mechanism of CHCHD2 in amyotrophic lateral sclerosis (ALS) by establishing a cellular model of the P14L CHCHD2 mutation and a Drosophila model. They found that the P14L mutant leads to the mislocalization of CHCHD2 to the cytoplasm, which affects calcium (Ca^2+^) uptake by mitochondria. However, due to the limited number of studies, the accuracy of this hypothesis requires further validation.

### 3.3 CHCHD2 and the treatment of neurodegenerative diseases

As research on CHCHD2 deepens, researchers have increasingly recognized its potential as a therapeutic target for neurodegenerative diseases. They have identified various therapeutic approaches to mitigate its loss-of-function effects or inhibit its gain-of-function mechanisms. Given that CHCHD2 deficiency is closely associated with mitochondrial dysfunction, mitochondria-targeted therapies may partially alleviate the mitochondrial impairments caused by CHCHD2 mutations. Consequently, many researchers have begun to explore treatments for neurodegenerative diseases from the perspective of mitochondrial defects, with or without CHCHD2 mutations, in an effort to discover new therapeutic strategies.

Initial studies have shown that overexpression of CHCHD2 in a cellular model of mitochondrial disease can rescue the mitochondrial phenotype, demonstrating its therapeutic potential as a transcriptional activator (Aras et al., 2020). In contrast to the mutant form, CHCHD2 promotes the assembly of the F1F0-ATPase, an enzyme critical for intracellular ATP synthesis, thus preserving its function and activity to alleviate mitochondrial dysfunction in Parkinson’s disease (PD; Chen et al., 2024). Additionally, CHCHD2 plays a cytoprotective role by maintaining mitochondrial homeostasis and promoting neuronal proliferation (Li et al., 2022). In a cellular model of Huntington’s disease, overexpression of CHCHD2 corrected mitochondrial defects, underscoring its potential as an early intervention target for this condition (Lisowski et al., 2024).

In 2004, Zhao et al. developed Elamipretide, a cell-permeable and mitochondria-targeted peptide antioxidant aimed at the inner mitochondrial membrane (IMM; Zhao et al., 2004). This peptide was shown to reduce cell death induced by mitochondrial reactive oxygen species (ROS) production (Zhao et al., 2005), thereby demonstrating its therapeutic potential for aging and oxidative stress-related diseases. Subsequent studies indicated that Elamipretide improved symptoms in conditions such as Barth syndrome (Thompson et al., 2024) and mitochondrial myopathy (Karaa et al., 2020), and was able to reverse mitochondrial defects, which is expected to be a new therapy directly targeting age-related mitochondrial dysfunction (Roshanravan et al., 2021). Furthermore, Elamipretide may benefit patients with Parkinson’s disease (PD) associated with CHCHD2 mutations, as found by Zhou et al. (2019) in neural progenitor cells carrying the R145Q mutation. This research demonstrated that Elamipretide could attenuate R145Q-induced mitochondrial dysfunction by protecting mitochondrial cristae. In the same year, Lmai et al. (2019) identified a light-driven proton transporter protein, a mitochondria-targeted Delta-retinal, that protected mitochondrial function. This protein alleviated cellular defects caused by CHCHD2 deficiency through light-dependent activation, effectively reversing the pathology associated with CHCHD2 deficiency. Both findings highlight therapeutic strategies targeting the consequences of disrupted CHCHD2 function. Regarding the inhibition of CHCHD2’s toxic function, Tio et al. (2024) discovered a protein, P32, which interacts with CHCHD2. Knocking down P32 reduced CHCHD2 expression in a CHCHD2-Arg145Gln mutant model and alleviated the PD-associated phenotype caused by the mutation, while protein expression remained unaffected in models expressing wild-type CHCHD2. This suggests that deletion of P32 inhibits the expression of mutant CHCHD2 and its downstream pathway activation, thereby reducing the toxic gain of function associated with the mutation and ameliorating the PD-associated phenotype. Although the precise molecular mechanism remains unclear, this finding opens new avenues for future research into the treatment of CHCHD2-associated neurodegenerative diseases.

## 4 Summary and outlook

CHCHD2, a mitochondrial regulatory factor, plays a crucial role in mitochondrial homeostasis and function and is strongly linked to neurodegenerative diseases. It is essential for maintaining normal mitochondrial function while also promoting tumorigenesis, suggesting a potential correlation between these two roles. Pathogenic mutations in CHCHD2 have been implicated in various neurodegenerative disorders, underscoring its association with disease development. However, in view of the complexity of the pathogenesis of neurodegenerative diseases, which may involve multiple mechanisms, it is not possible to attribute it to a single gene mutation. The lack of identified pathogenic mutations in some studies further supports this perspective. Although numerous studies have highlighted the involvement of CHCHD2 mutations in disease progression, the precise molecular mechanisms underlying these associations are not yet fully understood. Consequently, extensive research is necessary for further validation and refinement. Additionally, CHCHD2 is being explored as a potential therapeutic target for neurodegenerative diseases, particularly in terms of loss-of-function and gain-of-function mechanisms. However, the limited body of literature on this topic necessitates additional studies for future validation. Investigating how to integrate these two therapeutic approaches to achieve optimal outcomes also warrants further consideration.
